# Personalizing the Treatment of Women with Ductal Carcinoma In Situ (DCIS) Using the DCIS Score: A Qualitative Study on Score Use

**DOI:** 10.3390/curroncol31020073

**Published:** 2024-02-10

**Authors:** Mary Ann O’Brien, Lawrence Paszat, Tutsirai Makuwaza, Cindy Fong, Eileen Rakovitch

**Affiliations:** 1 Department of Family and Community Medicine, University of Toronto, 500 University Avenue, 5th Floor, Toronto, ON M5G 1V7, Canada; 2 Sunnybrook Health Sciences Centre, Sunnybrook Research Institute, 2075 Bayview Avenue, Toronto, ON M4N 3M5, Canada; lawrence.paszat@sunnybrook.ca (L.P.); eileen.rakovitch@sunnybrook.ca (E.R.); 3 Independent Researcher, Hamilton, ON L8K 1T9, Canada; 4 ICES, V1 06, 2075 Bayview Avenue, Toronto, ON M4N 3M5, Canada; cindy.fong@ices.on.ca

**Keywords:** decision making, carcinoma, intraductal, noninfiltrating, radiation oncology, implementation science

## Abstract

Background: A twelve-gene molecular expression assay (DCIS score) may help guide radiation oncology treatment under specific circumstances. We undertook a study to examine radiation oncologist (RO), surgeon, and decision maker views on implementing the DCIS score in practice for women with low-risk DCIS. Methods: We conducted a qualitative study involving telephone interviews that were audio-recorded and transcribed. Two researchers conducted a thematic analysis of transcripts. Results: Twenty-eight individuals (ROs, breast cancer surgeons, and cancer policy decision makers) were invited to participate; 22 out of the 28 people (79%) agreed. The final sample included 20 participants: 11 of 13 (85%) ROs, 5 of 7 (71%) surgeons, and 4 of 8 (50%) decision makers. Most ROs expressed concerns about overtreatment but could not predict with certainty which low-risk patients could safely avoid radiation. The DCIS score was viewed as contributing valuable personalized risk information as part of treatment decision making that included clinicopathological factors and women’s preferences. Future implementation would require guidelines with input from the oncology team. Conclusions: ROs had concerns about the overtreatment of women with DCIS, but lacked the tools to reliably predict which women could safely avoid radiation. By providing oncologists and women with personalized tumor information, the DCIS score was an important component of treatment decision making.

## 1. Introduction

The Oncotype DCIS score is determined from a multigene expression assay performed on RNA that was extracted from tissue blocks containing ductal carcinoma in situ (DCIS) resected during surgery [1]. The test generates a score of 0–100, based on 12 specific genes in the breast tumor tissue. The DCIS score is classified into a low, intermediate, or high risk of recurrence; a low-risk score is less than 39; a high-risk score is 55 or higher; and a score of 39 to 54 is intermediate risk [2,3]. Evidence is emerging that the DCIS score may be useful in guiding radiation oncology treatment under specific circumstances [2,3,4]. In 2018, Paszat et al. demonstrated that it is possible to calculate an individual woman’s risk of recurrence after breast conserving surgery using the DCIS score and clinicopathological risk factors [5]. Among a population of 1102 women with DCIS in Ontario, Canada, the range of the predicted risks of recurrence using the DCIS score was much wider than the range of risks calculated without the DCIS score [5]. Moreover, among women who would usually have radiation, the study results suggested that a low DCIS score (less than 39) could avoid overtreatment with radiation, sparing these women from radiation side effects. The DCIS assay costs about USD 3400 and is being used in the United States (US), where the cost is much less than the cost of breast radiotherapy, previously estimated as USD 10,500 [6]. The cost of this assay may be offset in Ontario by avoiding radiotherapy for a significant number of women with very low risks of local recurrence, who might otherwise have received it [7]. Currently, the DCIS assay is not funded in Ontario.

Members of our research team recently explored the use of the DCIS score by radiation oncologists and their patients in making decisions about radiation therapy post breast conserving surgery for women diagnosed with DCIS and without adverse clinicopathological features [8]. The ‘DUCHESS’ study found that the use of the DCIS score combined with clinicopathological features identified more women with a predicted low (<10%) 10-yr local recurrence risk after breast conserving surgery, leading to a significant decrease in recommendations for radiation therapy when compared with recommendations based on clinicopathological factors alone [8]. Considering these results, we undertook a follow-up qualitative research study of DUCHESS to understand the following:

- Radiation oncologist and breast cancer surgeon views on the contribution of the DCIS score for assessing the risk of local recurrence and the avoidance of radiation therapy post breast conserving surgery for women with a confirmed diagnosis of DCIS and without adverse clinicopathological features.
- Cancer policy decision-maker views on barriers and facilitators to funding the DCIS score for women with a confirmed diagnosis of DCIS and without adverse clinicopathological features to avoid overtreatment with radiation therapy.

The results of this study could inform the future implementation of the DCIS score in clinical practice.

## 2. Materials and Methods

Additional information about the study methods is found in the Supplemental Materials, Table S1: Consolidated criteria for reporting qualitative studies (COREQ): 32-item checklist [9].

### 2.1. Sample

Radiation oncologists in academic and community cancer centres in Ontario were eligible to participate if they had enrolled patients in the DUCHESS study and provided treatment for women with DCIS. Our justification was that these oncologists, as part of the DUCHESS study, would have had clinical experience receiving the Oncotype DCIS score results and the opportunity to use these results in their decision making about radiation therapy. Because of their experience, we believed they could provide in-depth data which could then contribute to the knowledge regarding future test implementation in actual practice. Practicing breast cancer surgeons at academic and community centres in Ontario were also approached. Breast cancer surgeons were included because they are the clinicians who decide whether to refer patients with DCIS post breast conserving surgery to radiation oncologists for the consideration of radiation therapy. We believed that breast cancer surgeons were important to include because of treatment planning discussions they have with patients which might influence patient’s expectations regarding subsequent radiation treatment. The third stakeholder group was composed of cancer policy decision makers at the cancer system organization for the province (Ontario Health/Cancer Care Ontario), who had responsibilities in treatment programs for DCIS and who advise the Ministry of Health on policy and resource allocation. We included this group because they were knowledgeable about how the provincial government makes funding decisions regarding molecular biomarker assays. The input by cancer policy decision makers could help inform the future implementation of such assays for DCIS. We used purposeful sampling to enroll clinicians [10,11] and snowball sampling to identify decision makers [10,11]. All participants were approached via email from the principal investigator (ER). We did not specifically recruit women with DCIS because other researchers have previously studied the views of women with DCIS [12]. Research Ethics Board approval was granted by the University of Toronto #00037939, Women’s College Hospital #2019-0010-E, and Sunnybrook Health Sciences Centre #2812.

### 2.2. Data Collection

An interview guide was created based on the study objectives and discussions among the study team, and then pilot tested with a practicing radiation oncologist who provided treatment for women with DCIS. Based on the pilot testing, several new questions were added and reviewed with the study team. Personal telephone interviews lasting approximately 25 min were conducted by a qualitative researcher (MAO) over 14 months. Interviews were audio-recorded, transcribed, and de-identified. We collected demographic information including age, sex, type of clinician, type of hospital, and years in practice.

### 2.3. Analysis

We used principles of grounded theory [13,14] and the constant comparative method to analyze the transcript data [13,14,15]. Two team members (MAO, TM) created a coding guide, then completed line-by-line coding on three transcripts; TM coded the remaining transcripts using the guide. MAO and TM identified the major themes in the coded data. Using the constant comparative method [13,14,15], we checked that the themes were supported by quotations across different types of participants (radiation oncologists, breast cancer surgeons, and policy decision makers when appropriate). We also noted when a quotation was not supportive of a theme. The study team reviewed the coded data, the major themes, and the supporting verbatim quotes. We used qualitative data management software (NVivo 11, QSR International (now Lumivero, Denver, CO, USA) to store the coded transcript data. We created an audit trail that documented all major analytic decisions [16]. We reached information data saturation after 18 interviews but included an additional two interviews to ensure that we had not inadvertently missed important information [17].

In qualitative research designs, the terms ‘trustworthiness’ or ‘credibility’ are often used to describe the rigor of research studies in place of ‘validity’ [18]. We ensured study credibility by substantiating all themes with textual data, reviewing codes, and themes with the team, and using qualitative data management software.

## 3. Results

Twenty-eight individuals (radiation oncologists, breast cancer surgeons, and decision makers) were invited to participate; twenty-two out of the twenty-eight agreed (79%), four did not reply, and two declined but provided alternative names. Twenty participants were interviewed (two individuals who had agreed did not respond to a request for an interview date). The participants included 11 of 13 (85%) radiation oncologists, 5 of 7 (71%) surgeons, and 4 of 8 (50%) decision makers. Of the twenty participants, eleven (55%) were female. Participants were an average age of 50, with an average of 17 years in clinical practice.

We identified seven themes derived from the interview data from radiation oncologists and surgeons on their perspectives of the DCIS score; we also present feedback from the cancer decision makers. See Table 1 for supporting quotations for each theme.

### 3.1. Concerns about the Overtreatment of Women with DCIS but Uncertain Which Low- Risk Patients Can Safely Avoid Radiation

Most radiation oncologists and surgeons expressed concerns about the overtreatment of women with low-risk DCIS. They reported difficulty knowing when DCIS is a serious disease and when it would have little clinical significance. Moreover, existing tools for risk prediction were described as inconsistent and oncologists expressed uncertainty in predicting which patients would have tumor recurrence and/or develop invasive cancer. While overall radiation was perceived as important for local control and for reducing the risk of recurrence—particularly since the research evidence demonstrates the benefits of radiation treatment and because radiation therapy is the standard of care—there was some concern that overtreatment may be occurring.

### 3.2. Patient Beliefs about DCIS and Their Experiences Prior to the Consultation Can Influence Their Expectations about Radiation Treatment

Radiation oncologists indicated that many patients do not have an accurate understanding of DCIS, and this inaccuracy may contribute to their expectations about the type of treatment that they will receive. For example, oncologists relayed that some patients believed they had invasive breast cancer which was perceived as life threatening. Patient misunderstandings about DCIS were attributed by oncologists to information that patients had received before the consultation. Several surgeons corroborated this finding by speculating that other clinicians may inadvertently convey ambiguous information to patients (e.g., describing DCIS as pre-breast cancer), leading to patient misconceptions about DCIS. Additional influences such as prior patient experiences with friends or family member treatment, exposure to public messaging about cancer (i.e., ‘war on cancer’), and previous discussions with their surgeon may have influenced patient expectations about radiation treatment.

### 3.3. The DCIS Score Was Positively Viewed as a Valuable Adjunct to Clinical Decision Making

According to radiation oncologists, the current tools for risk stratification lack consistency; oncologists said they are often unable to confidently identify which low-risk patients were likely to truly benefit from radiation treatment. Through experiences using the DCIS score during the DUCHESS study, oncologists perceived it as valuable by providing more individualized risk identification when compared to other tools and/or risk calculations based solely on clinicopathological factors. In addition, oncologists perceived that the DCIS test was more precise, thereby minimizing the uncertainty related to their own decision making and that of patients. The DCIS score supported the oncologists’ own treatment recommendations and the patients’ decisions as perceived by oncologists. They noted that while the score was often consistent with their assessment based on clinicopathological factors, there were also some surprises when reviewing the score, which impacted their treatment recommendations. In some cases, the score was higher than anticipated and resulted in the oncologist adding treatment or referring the patient back to the surgeon. While the score was perceived as useful for decision making for some patients, oncologists commented that every patient did not need the score for supported decision making. We found one case of a disconfirming view. For one oncologist, there were no surprises when using the DCIS score with patients and their perception was that it was not helpful for decision making.

### 3.4. The DCIS Score Was Helpful for Patients by Providing Personalized Information, Reassurance, and Help with Decision Making

Radiation oncologists noted that pre-invasive disease decisions, such as those for DCIS treatment, are more difficult for patients because radiation therapy does not have an impact on survival. Oncologists also described that patients may have different perceptions of risk compared to oncologists, such that some patients perceive themselves to be at a greater risk than the clinical data would suggest. Patients were perceived to have more difficulty with decisions when clinicopathological factors indicated uncertainty with respect to treatment.

Oncologists perceived that the DCIS score helped low-risk patients who were nervous about radiation to avoid it. In other scenarios, oncologists reported it helped reassure patients—for example, it was helpful for undecided patients, and often convinced them to proceed with radiation because their risk score was high. Similarly, it helped reassure patients about their low risk (the score showed risk was not as high as the patient perceived), even though some opted to proceed with the radiation. Oncologists described that most women were open to using the DCIS score to obtain additional information, reassurance, and help in decision making.

### 3.5. Surgeons Had Mixed Views about Who Should Order the DCIS Test in Future Implementation

Surgeons had mixed views regarding the responsibility for ordering the DCIS test in future clinical implementation. Some surgeons reported that they would not order the test themselves, and instead firmly viewed this role and that of interpreting the test results as belonging to radiation oncologists. One surgeon recommended that protocols for ordering the DCIS test should follow those currently in place for the Oncotype DX score for invasive breast cancer; hence, it should be oncologist driven. Furthermore, surgeons indicated that, since radiation can be administered within the 12 weeks following breast conserving surgery, there was not an urgent need for surgeons to order this test. Instead, surgeons said that the patient should consult with the radiation oncologist who is best qualified to decide which patient could benefit from the DCIS test. Some surgeons were willing to order the DCIS test with an added caveat: they would need to know that the radiation oncologist would find the results useful. They suggested that if surgeons ordered the test prior to the radiation oncology consultation, it would set the stage for the next appointment, thus preparing patients for future DCIS treatment discussions with the radiation oncologist.

### 3.6. Barriers to Implementing the DCIS Score in Clinical Practice

Insufficient evidence for the validation of the DCIS score was seen as a barrier to implementing the DCIS score in practice, both by radiation oncologists and surgeons. The costs and lack of government funding for the test in Canada were also mentioned as key barriers by both oncologists and surgeons.

Three other barriers to score use in practice were noted by radiation oncologists: a time-intensive, physician ‘bias’ in favour of radiation therapy, and a lack of education regarding the score. Using the score was perceived as time-intensive, as it involved an extra visit. With respect to physician ‘bias’, some oncologists may believe that radiation therapy is unlikely to harm patients with DCIS and is more likely to be beneficial by lowering the risk of local recurrence. Lastly, oncologists reported a need for additional education and training for better understanding of the score.

Surgeons were primarily concerned about the implications for radiation oncologists with respect to additional workload. Surgeons also perceived that a lack of standardization, including a lack of consensus among radiation oncologists on appropriate test use and a lack of treatment algorithms for surgeons, could be barriers to using the test. Without standardization, surgeons may be reluctant to mention this test to patients, as it could inadvertently put their oncologist colleagues “in a corner”.

### 3.7. Facilitators to Implementing the DCIS Score in Clinical Practice

Radiation oncologists highlighted several facilitators to using the DCIS score in practice. These were as follows: the importance of clinical team buy-in and ensuring a multi-disciplinary approach, prior to implementing the test as standard practice; more experience with score interpretation; and the development of guidelines for test use. Oncologists indicated that practice tools should not be tedious or time-consuming, and the testing process could be streamlined to save patient and oncologist time.

Surgeons supported the need for additional rigorous research evidence for using the DCIS score. In addition, surgeons wanted feedback from radiation oncologists, indicating the score as acceptable and useful prior to implementation. Surgeons perceived that using the DCIS score would influence a change in practice and would gradually become part of the treatment for women with low-risk DCIS, like OncotypeDx, for invasive breast disease.

## 4. Feedback from Cancer Policy Decision-Makers

Feedback from cancer decision makers in Ontario provided insight on the different ways in which evidence for new cancer technologies such as the DCIS test is reviewed and considered for province-wide implementation. The feedback suggests that the emerging evidence needs to be considered a priority and must be of high quality, showing clinical benefits for patients. Evidence synthesis was seen as an important first step of implementation, and decision makers described specific health agencies that synthesize and rigorously examine study data. Cost effectiveness and the potential to save resources or reduce hospital wait-times were important considerations for recommending the uptake of new interventions. Decision makers also supported the development of guidelines for using the DCIS test, and suggested all stakeholders (radiation oncologists, breast cancer surgeons, and pathologists) should be engaged in discussions about implementation.

## 5. Discussion

This study provides information about several barriers and facilitators to implementing a new cancer technology (DCIS score) in practice, as perceived by radiation oncologists, breast cancer surgeons, and decision makers in Ontario. The study highlighted that radiation oncologists and breast cancer surgeons perceive that radiation therapy is accepted as the standard of care for the treatment of women with DCIS, but there are concerns about the potential for overtreatment and exposure to the potential toxicities of radiation in women without adverse clinicopathological features. Radiation therapy can adversely affect quality of life during treatment with patients reporting fatigue and breast pain [19]. The serious late effects of radiation can include skin changes (e.g., hyperpigmentation, fibrosis) and a small increased risk of rib fractures. The rare but possible life-limiting effects of radiation include second malignancies [20] and a potential increased risk of cardiac disease [21]. Moreover, breast radiation therapy is inconvenient for the patient, as it is administered daily for up to 3½–6 weeks and must be given at a limited number of accredited radiotherapy centres in Canada, so that patients must often travel long distances for treatment. Radiation oncologists in our study described the difficulties in determining which low-risk patients can safely avoid radiation because existing risk assessment tools are inconsistent and imprecise. The DCIS score was positively valued by oncologists as an additional tool to assess risks, as it provided personalized information for both oncologists and women, which helped with decision making and reassured clinicians about their treatment recommendations.

Oncologists perceived that the DCIS score provided more granular, tangible information about the recurrence risk, which helped women in deciding whether to forgo or choose radiation and reassured them about their treatment choices. Oncologists described women as often feeling overwhelmed or conflicted by decision making, and the DCIS score was perceived as a helpful tool. Other researchers have found that women with DCIS had similar serious concerns about their risk of recurrent disease and expressed uncertainty about treatment decision making [12]. In our current study, oncologists also noted that women’s beliefs about DCIS, prior conversations with their family physician or breast cancer surgeon, and women’s tolerance of risks contributed to women’s expectations around the treatment. Some patients wanted to avoid radiation, while some sought every possible therapy despite low-risk DCIS. The DCIS score was therefore helpful in explaining risks to patients, providing them with pertinent information and guiding conversations around treatment decision making, which supports findings from recent research [22,23].

Although the clinical benefits of the DCIS score for low-risk patients have been previously reported [1,2,3,4,5,7,8], the process of clinical practice implementation is not well known. The decision makers interviewed in this study outlined how evidence synthesis is the first step to uptake. Subsequently, key clinical leaders should alert policymakers of new practice-changing evidence. All participants in our study indicated that important facilitators for clinical uptake were the evidence of cost effectiveness and clinical utility. Other considerations were the potential for additional oncologist workload and extra time requirements for patients who received the test. Identified facilitators to clinical uptake were establishing guidelines and standardization on the use of the DCIS score for a unified understating as to which patients could benefit from DCIS testing and who would order the test and interpret the findings. Our findings are consistent with the Consolidated Framework for Implementation Research, which identified five broad dimensions as potentially important for the implementation of innovations [24]. The importance of patient benefits and input from a multidisciplinary team were especially relevant.

In summary, our study is an early examination of the implementation of a specific molecular biomarker (Oncotype DCIS) under particular circumstances in one large Canadian province. Our early findings may have implications for radiation oncology leaders, for breast disease teams, and for Ontario Health/Cancer Care Ontario. When there is sufficient evidence to warrant implementation, leaders will need to ensure the training of radiation oncologists regarding test use and interpretation. The multidisciplinary team will need to decide on roles and responsibilities for ordering the test and the communication of the results to patients. The team should ensure consistent messages are given to patients by breast cancer surgeons and by oncologists regarding radiation treatment planning in order to avoid inadvertent misunderstanding by patients. Lastly, for Ontario Health/Cancer Care Ontario, once there is sufficient evidence for test implementation, clinical practice guidelines will need to be created and disseminated.

### Study Limitations and Strengths

We interviewed only two radiation oncologists and one surgeon from community hospitals, and it is uncertain if our research findings would apply to clinicians treating patients with DCIS in community hospital settings. Moreover, radiation oncologists in our study reflected on their experiences using the DCIS score in the context of the DUCHESS study, rather than its use in routine clinical practice. We do not know if our findings would be similar to those found in clinical practice. We did not explicitly include medical oncologists in our sample. Their inclusion could have provided insights into the adoption of other molecular biomarker assays, such as Oncotype DX for adjuvant chemotherapy decisions in breast cancer clinical practice. We also recognize that there is another molecular biomarker assay (DCISionRT Decision Score (Prelude DX)) which can predict risk recurrence after breast conserving surgery for DCIS [25]. However, since the twelve-gene Oncotype DCIS assay was used in the parent DUCHESS study, we focused our efforts on understanding radiation oncologist experiences with this specific assay. A study strength was that we recruited radiation oncologists and surgeons from academic hospitals at different centres and with considerable experience in clinical practice (on average, 17 years). We recruited decision makers with an average of nine years of experience, which provided insights into future implementation. We reached informational saturation [17] during the analysis of data.

## 6. Conclusions

Radiation oncologists in this qualitative research study had concerns about the overtreatment of women with DCIS, but lacked tools to reliably predict which women could safely avoid radiation. By providing oncologists and women with personalized tumour information, the DCIS score was an important component of treatment decision making for both oncologists and women. Involvement in planning by the oncology team was seen as an essential facilitator to the future implementation of the DCIS test in clinical practice.

## Figures and Tables

**Table 1:** Main themes and exemplar supporting quotations.

	Theme	Exemplar Supporting Quotations
1.	Concerns about the overtreatment of women with DCIS [Ductal Carcinoma in Situ] but uncertain which low-risk patients can safely avoid radiation.	*“I think we definitely over-treat. … We are over-treating but we don’t have a good gauge of which patients we can forgo treatment. You know, even in the lowest risk categories, we still have reduction risk around 10%.”* (Radiation Oncologist (RO2) ^1^ *“I do look at all the risk factors and pathological factors to try to help guide the indication for radiation. As we know, many of the randomized trials haven’t really shown a clear subset that don’t need radiation or a clear subset of patients that would do well without radiation.“* (RO4) *“I think we’re more towards recommending radiation than not. So we tend towards the over-treatment side.”* (RO6)
2.	Patient beliefs about DCIS and their experiences prior to the consultation can influence their expectations about treatment.	*“Some women say, “Well, my surgeon told me that I needed radiation.”… And so they come expecting that, well, their surgeon said they needed radiation so they need radiation.”* (RO10) *“…it ties in with the war on cancer that is now being replaced by the war on terrorism, but it was the war on cancer that you had to do everything and anything at all cost to fight breast cancer.”* (RO3) *“So I would say it really depends who they’ve seen before they see me. So if they’re coming from a family doctor or anyone else, say their ob/gynae referred them to me, and they’ve got DCIS …they don’t realize the difference between cancer and DCIS. I had a lady in clinic yesterday, and, she had low grade DCIS, and she thought she had cancer.”* (Surgeon (S)2)
3.	The DCIS score was positively viewedas a valuable adjunct to clinical decision making.	*“It isn’t the only thing* [DCIS score] *we should be looking at but it adds to the overall decision-making.”* (RO7) *“And there were two or three patients where it made a difference. I think it was to confirm not to go ahead with the radiation in two, and one to give radiation.”* (RO13) *“That was another advantage of the Oncotype. Because sometimes I had tests that came back really, really high. Like super high. And in those cases, I did seek further assessment. Like either with chemo prevention or giving more radiation dose.”* (RO5)
4.	The DCIS Score was helpful for patients by providing personalized information, reassurance, and help with decision-making.	*“…in my practice, they [patients] felt much more secure when they saw the score or it aligned with it, or just helped confirm what they were already comfortable doing … So yeah, overall it was extremely helpful.”* (RO7) *“... the more information you can give patients that help them either make the decision or be comfortable with the decision that they will have made, it ends up just being better for kind of quality of life and I suspect long term acceptance of whatever outcomes they have because they made the decision based on as good evidence as they could have.”* RO1
5.	Who should order the DCIS test in future implementation?	*“I mean I think it would be the rad oncs who would order it. So the rad oncs would have to buy into it.”* (S2) *“The judgment as to whether or not a patient would benefit from the test and determine whether they would benefit from radiation is up to the radiation oncologists.”* (S3) *“And again, it’s hard to convince people to go to an oncology appointment. I’m like, “Go. And don’t worry, there’s a score that predicts if you need… the treatment, or not.” So they’re like, “Yeah, but I only want to do it if I had to do it,” is generally the women’s response to adjuvant care. And so then it’s a lot easier for the surgeon to say, “Well, I’ll order it and that way when you go to that appointment, they’ll have the score and they’ll be able to say for you specifically if it’s useful or not useful.”* (S4)
6.	Barriers to implementing the DCIS score in clinical practice	
	(a) Radiation oncologist views of the barriers	*“…the only barrier is time. The barrier is, now for instance, there is a test, I have to see the patient twice.”* (RO8)
	(b) Surgeon views of the barriers	*“I’m sure not every radiation oncologist will want a test if it’s very clear in their mind that the patient needs to have radiation. And the opposite is true… It’s also putting the radiation oncologist as my colleague in a difficult situation if they didn’t think the test result was going to influence their decision….”* (S5) *“So we have to speak the same language. And so I wouldn’t do it if the rad oncs hated it or didn’t use it. But if it was useful or they didn’t really care, but it maybe helped the patients feel like it was a useful…like that it was a necessary treatment, then I would order it. But… So it has to be, I think, agreed upon by the group.”* (S3)
7.	Facilitators to implementing the DCIS score in clinical practice	
	(a) Radiation oncologist views	*“I guess the knowledge—like knowledge translation. So for better understanding. Because you don’t want a situation where people think it’s only about the score.”* (RO7) *“I think physicians are incredibly petty with their time. For good reason... So there has to be a way to have it* [DCIS score] *streamlined. I think ideally if Oncotype became a standard of care for patients with DCIS, ideally what would happen is the DCIS testing would be done prior to being seen.”* (RO1)
	(b) Surgeon views	*“I mean I think the science would have to be good so that the radiation oncologists felt comfortable with doing it.”* (S2)
8.	Cancer Policy Decision-Maker Views	*“So I would say it has to be rigorous evidence. And then the other thing would be the cost and whether it’s cost effective.”* (Decision-Maker (DM)2) *“Well, one* [facilitator to implementation] *would be evidence of clinical benefit. The ability to save toxicity for the patient. So omitting radiation therapy without any further consequences in terms of recurrence or death. That would be one reason. The other would be cost effectiveness.*” (DM3) *“But, you know, it’s the cost of implementing an extra step upfront to potentially save money later, I think is what makes everyone hesitate. So if you say, oh, we have a new test at $5000 US, everyone just sees the $5000 US, and they don’t see potentially, $20,000 or whatever it is for the cost of radiation therapy that could be saved. So I think that’s the main thing.“* (DM4) *“…it would be nice to involve surgeons, oncologists and pathologists. But I really think it’s going to be a rad onc-driven test. And then everyone else is kind of, you know, it would be nice to have their input as well. But I think it’s mainly the rad onc people”* (DM4)

^1^ Refers to the participant number.

## Data Availability

The interview transcripts are not available because participants did not consent to publication of the transcripts when they agreed to participate in the study.

## Supplementary Materials

The following supporting information can be downloaded at: https://www.mdpi.com/article/10.3390/curroncol31020073/s1, Table S1: Consolidated criteria for reporting qualitative studies (COREQ): 32-item checklist.

## Abbreviations

DCIS	Ductal Carcinoma in Situ
DCIS score	OncotypeDX Breast DCIS Score Test®
DUCHESS	Evaluation of the DCIS score for Decisions on Radiotherapy for Patients with Low/Intermediate Risk DCIS
KT	Knowledge translation
