# Supplementary material for: Personalizing the Treatment of Women with Ductal Carcinoma In Situ (DCIS) Using the DCIS Score: A Qualitative Study on Score Use
Source: Curr Oncol. 2024 Feb 10;31(2):975–86. doi: 10.3390/curroncol31020073 (PMC10887948; doi:10.3390/curroncol31020073)
Supplement: Supplementary file 1 [file curroncol-31-00073-s001.zip › curroncol-2711960-supplementary.pdf]

Personalizing the Treatment of Women with Ductal Carcinoma in Situ (DCIS) Using the DCIS Score: A Qualitative Study on Score Use

Table S1. Consolidated criteria for reporting qualitative studies (COREQ): 32-item checklist (Tong et al. 2007)

Domain	Item	Author's Response
**1. Research team and reflexivity**	**Personal Characteristics**<br>1. **Interviewer/facilitator**<br>Which author/s conducted the interview or focus group?	Interviews were conducted by Mary Ann O'Brien (MAO), PhD.
	**Credentials**<br>2. What were the researcher's credentials? E.g., PhD, MD	MAO's credentials: PhD
	3. **Occupation**<br>What was their occupation at the time of the study?	MAO is a researcher in the Department of Family and Community Medicine, University of Toronto.
	4. **Gender**<br>Was the researcher male or female?	MAO identifies as female.
	5. **Experience and training**<br>What experience or training did the researcher have?	MAO and Tutsirai Makuwaza (TM) have received training in qualitative research methods and have conducted qualitative research studies.
	**Relationship with participants**<br>6. **Relationship established**<br>Was a relationship established prior to study commencement?	Neither MAO nor TM had a relationship with study participants prior to the study.

	7. **Participant knowledge of the interviewer**<br><br>What did the participants know about the researcher (e.g., personal goals, reasons for doing the research)?	The participants did not have any detailed information about the interviewer. MAO introduced herself at the beginning of the interview.
	8. **Interviewer characteristics**<br><br>What characteristics were reported about the interviewer/facilitator (e.g., bias, assumptions, reasons, and interests in the research topic)?	MAO was described as an experienced qualitative researcher.
**2. Study design**	**Theoretical framework**<br><br>9. **Methodological orientation and theory**<br><br>What methodological orientation was stated to underpin the study (e.g., grounded theory, discourse analysis, ethnography, phenomenology, content analysis)?	In the analysis, we used the constant comparative method which has its roots in grounded theory.
	**Participant selection**<br><br>10. **Sampling**<br><br>How were participants selected (e.g., purposive, convenience, consecutive, snowball)?	For the radiation oncologists and surgeons, we used purposive sampling as follows: to be considered for inclusion in the study, radiation oncologists had to have participated in the DUCHESS parent study in Ontario, Canada. Breast cancer surgeons had to provide care in Ontario for women with DCIS. For the decision-makers, we used snowball sampling whereby decision-makers who

		agreed to participate in the study provided suggestions for other decision-makers. In addition, decision-makers had to have a role in the provincial cancer system.
	11. **Method of approach**<br><br>How were participants approached (e.g., face-to-face, telephone, mail, email)?	Participants were approached by email from Dr. E Rakovitch.
	12. **Sample size**<br><br>How many participants were in the study?	Twenty participants were interviewed.
	13. **Non-participation**<br><br>How many people refused to participate or dropped out, and what were their reasons?	Initially, twenty-eight people were invited to participate. Four participants did not reply, two refused but provided additional names. Subsequently, two other participants who had agreed to participate did not respond to a request for an interview date, leaving twenty participants who were interviewed.
	**Setting**<br><br>14. **Setting of data collection**<br><br>Where was the data collected (e.g., home, clinic, workplace)?	Data were collected by telephone.
	15. **Presence of non-participants**<br><br>Was anyone else present besides the participants and researchers?	No one else was present during the interviews.

	16. Description of sample<br><br>What are the important characteristics of the sample (e.g., demographic data, date)?	Participants included 11 of 13 (85%) radiation oncologists, 5 of 7 (71%) surgeons, and 4 of 8 (50%) decision-makers. Of the twenty participants, eleven (55%) were female. Participants were an average age of 50, with an average of 17 years in clinical practice.
	17. **Interview guide**<br><br>Were questions, prompts, guides provided by the authors? Was it pilot tested?	We created an interview guide that was pilot-tested with a radiation oncologist who was not part of the study.
	18. **Repeat interviews**<br><br>Were repeat interviews carried out? If yes, how many?	There were no repeat interviews.
	19. **Audio/visual recording**<br><br>Did the research use audio or visual recording to collect the data?	We used audio recordings.
	20. **Field notes**<br><br>Were field notes made during and/or after the interview or focus group?	We created field notes during and after each interview.
	21. **Duration**<br><br>What was the duration of the interviews or focus group?	Each interview was approximately 25 min.
	22. **Data saturation**<br><br>Was data saturation discussed?	We reached data saturation after 18 interviews but held another 2 interviews to ensure that no major areas were missed.

	23. **Transcripts returned**<br><br>Were transcripts returned to participants for comment and/or correction?	Transcripts were not returned to participants. Each transcript was checked for accuracy by MAO.
**3. Analysis and findings**	**Data analysis**<br><br>24. **Number of data coders**<br><br>How many data coders coded the data?	There were 2 data coders (MAO and TM).
	25. **Description of the coding tree**<br><br>Did authors provide a description of the coding tree?	We did not provide a description of the coding tree, but it is available from the authors.
	26. **Derivation of themes**<br><br>Were themes identified in advance or derived from the data?	Themes were identified inductively from the data.
	27. **Software**<br><br>What software, if applicable, was used to manage the data?	NVivo 11 (QSR International, now Lumivero) was used for data management.
	28. **Participant checking**<br><br>Did participants provide feedback on the findings?	Participants did not provide feedback on the findings.
	**Reporting**<br><br>29. **Quotations presented**<br>Were participant quotations presented to illustrate the themes / findings? Was each quotation identified?, e.g., participant number	Supporting quotations were provided to illustrate the themes (Table1). Each quotation was identified using a participant number.

	30. **Data and findings consistent**<br><br>Was there consistency between the data presented and the findings?	We believe that there is consistency between the data and the findings.
	31. **Clarity of major themes**<br><br>Were major themes clearly presented in the findings?	We believe that the major themes are clearly presented.
	32. **Clarity of minor themes**<br><br>Is there a description of diverse cases or discussion of minor themes?	We believe that the minor themes are clearly presented. We noted when there was a disconfirming view.

Reference

Tong, A.; Sainsbury, P.; Craig, J. Consolidated criteria for reporting qualitative research (COREQ): A 32-item checklist for interviews and focus groups. *Int. J. Qual. Health Care* **2007**, *19*, 349–357.
